# Light-Emitting Devices Based on Top-down Fabricated GaAs Quantum Nanodisks

**DOI:** 10.1038/srep09371

**Published:** 2015-03-20

**Authors:** Akio Higo, Takayuki Kiba, Yosuke Tamura, Cedric Thomas, Junichi Takayama, Yunpeng Wang, Hassanet Sodabanlu, Masakazu Sugiyama, Yoshiaki Nakano, Ichiro Yamashita, Akihiro Murayama, Seiji Samukawa

**Affiliations:** 1 World Premier International Center Initiative Advanced Institute for Materials Research, Tohoku University, 2-1-1 KatahiraAoba-ku, Sendai, Japan; 2 Graduate School of Information Science and Technology, Hokkaido University, Kita 14, Nishi 9, Kita-ku, Sapporo, Japan; 3 Institute of Fluid Science, Tohoku University, 2-1-1 KatahiraAoba-ku, Sendai, Japan; 4 School of Engineering, The University of Tokyo, 7-3-1 HongoBunkyo-ku, Tokyo 113-8656, Japan; 5 Research Center for Advanced Science and Technology, The University of Tokyo, 4-6-1, KomabaMeguro-ku, Tokyo, Japan; 6 Graduate School of Materials Science, Nara Institute of Science and Technology, 8916-5 Takayama, Ikoma, Japan; 7 CREST Japan Science and Technology Agency, 5 SanbanchoChiyoda-ku, Tokyo, Japan

## Abstract

Quantum dots photonic devices based on the III–V compound semiconductor technology offer low power consumption, temperature stability, and high-speed modulation. We fabricated GaAs nanodisks (NDs) of sub-20-nm diameters by a top-down process using a biotemplate and neutral beam etching (NBE). The GaAs NDs were embedded in an AlGaAs barrier regrown by metalorganic vapor phase epitaxy (MOVPE). The temperature dependence of photoluminescence emission energies and the transient behavior were strongly affected by the quantum confinement effects of the embedded NDs. Therefore, the quantum levels of the NDs may be tuned by controlling their dimensions. We combined NBE and MOVPE in a high-throughput process compatible with industrial production systems to produce GaAs NDs with tunable optical characteristics. ND light emitting diode exhibited a narrow spectral width of 38 nm of high-intensity emission as a result of small deviation of ND sizes and superior crystallographic quality of the etched GaAs/AlGaAs layer.

Quantum dots (QDs) have attracted considerable interests due to their potential device applications for the next generation quantum cryptosystem such as single-photon source, light emitting diodes, detectors, and laser diodes^1^. The active gain medium of QD semiconductor has numerous merits that are not available in a form of bulk or quantum wells^2^,^3^. For example, high-speed modulation, low-power consumption, and temperature independence have been demonstrated based on the QD technology^4^,^5^. Current research efforts focus on the lattice-mismatched QD systems such as InAs/GaAs grown in Stranski–Krastanov (SK) mode. While these systems are promising for application to telecommunication devices in the near-infrared wavelength range^4^,^5^,^6^, the structures are usually complex due to the strained, heavy intermixing, and wetting layers (WLs)^7^,^8^. Besides, the WLs under QDs behave as a carrier trap layer for QDs. These structures make noncontiguous energy bands drop to the quantum energy level of QDs, and hence the carrier relaxation is strongly affected by the ground states of QDs^9^, obscuring the intrinsic properties of QD devices. Due to these fundamental reasons and also to the indention to create prototype devices, it is desirable to develop a GaAs/AlGaAs system in a lattice-matched condition, where strain-free QDs without WLs can be formed as an ideal quantum structure. To this end, we have investigated a GaAs/AlGaAs system by using the ultimate top-down technique^10^, and recently observed a photoluminescence (PL) emission from the GaAs/AlGaAs nanodisks (NDs)^11^,^12^.

Reactive ion etching (RIE) is conventionally used to pattern optoelectronic devices without the active layer such as nanowire waveguide, distributed Bragg reflector (DBR), and distributed feedback (DFB) structures. On the other hand, RIE process for the quantum nanostructures of III-V compound semiconductor still remains inapplicable due to the generation of defects after the irradiation of charged particles and the vacuum ultraviolet (UV) photons. The RIE damage in GaAs is known to penetrate to a depth of several tens of nanometers from the surface.

Here we report the successful demonstration of a current-injected LED that was developed by the top-down process on the GaAs quantum NDs. As a result, we experimentally found a narrow line-width emission, reflecting the small distribution of the NDs sizes. The LED operation was confirmed at a low temperature of 7 K.

The sample was grown on a GaAs (001) substrate using the metal-organic vapor phase epitaxy (MOVPE). At first, MQWs structure consisted of a 100-nm-thick Al_0.15_Ga_0.85_As and MQWs of three pairs of 8-nm-thick GaAs well and 12-nm-thick Al_0.15_GaAs barrier for PL measurement, a single QW structures consisted of a 100-nm-thick Al_0.25_Ga_0.75_As and QW of a 4-nm- and an 8-nm-thick GaAs well, a 20-nm-thick Al_0.275_GaAs barrier.and 5-nm-thick capping layer for high energy level off-set for strong quantum energy level confinement for various temperature measurement, and MQWs device structure consisted of a 1.4-μm-thick Al_0.35_Ga_0.65_As bottom cladding layer and a 100-nm-thick Al_0.25_Ga_0.75_As separated confinement heterostructure (SCH) layer, and MQWs of six pairs of 8-nm-thick GaAs well and 12-nm-thick Al_0.15_GaAs barrier. The NDs in the active layer were formed by the ultimate top-down combination processes of the bio-template and the neutral beam (NB) etching for LED^13^,^14^,^15^,^16^.

Figure 1 shows a schematic diagram of the fabrication process, which includes nanopatterning using nanopillars prepared through NBE and regrowth using MOVPE. At first, the sample was cleaned using organic treatment, followed by deionized water in an ultrasonic bath to make the surface hydrophilic. Ferritins which contains metal oxide cores and modified with polyethylene glycol (PEG ferritin), to be spaced at a distance greater than 30 nm, were used as a new biotemplate to eliminate the coupling of the wave functions between the GaAs NDs. The molecules were arranged on the GaAs by spin-coating at 500 rpm for 2 s and at 3000 rpm for 30 s. After spincoating, the arrangement of ferritins are shown as in Fig. 1 (b). The density of 1.1 × 10^11^ cm^−2^ is measured.

A super-molecule (protein) ferritin was used as an etching mask because of its molecule-level uniformity in both shape and size; a single ferritin molecule is made of an iron oxide core of 7 nm in diameter with an outer shell of protein of 12 nm^17^,^18^. A monolayer array of ferritin molecules was self-assembled onto the substrate surface. After the removal of the protein shell by oxygen annealing for 30 min at 350°C with an oxygen flow rate of 100 sccm and a process pressure of 32 Pa, an array of homogeneous nanoparticles of iron oxide was obtained. Hydrogen radical treatment was employed to remove the surface oxide.

As for NB etching, which is known as an advanced etching technique for a damage-free III-V compound semiconductor process, the apparatus consisted of a plasma chamber and a process chamber that were separated by a carbon aperture. The aperture was used to effectively neutralize the charged particles and to screen the UV photons from the plasma. Etching was performed by the NB without the usual damage from charged particles or high-energy UV photons. In particular, GaAs etching of high aspect ratio over 10 was achieved by using the iron oxide core as etching mask. After making the array of nanopillars, the iron cores were removed in a diluted hydrochloride acid. Finally, MOVPE regrowth process was performed to create an Al_0.15_GaAs barrier, followed by the MOVPE regrowth of a 20-nm-thick GaAs-cap for PL measurement. An Al_0.15_GaAs barrier, a 100-nm-thick Al_0.25_GaAs SCH, a 1-um-thick p-Al_0.35_GaAs, and a 20-nm-thick p-type GaAs cap for LED.

## Results

**Figure 2:**
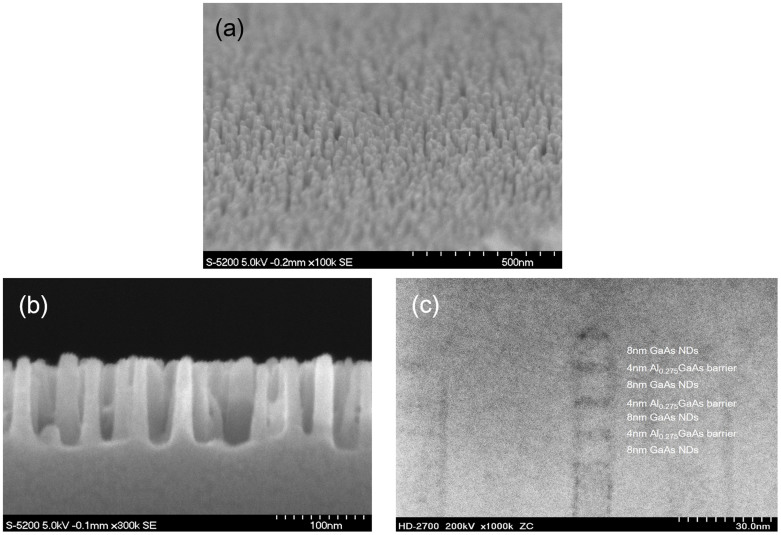
SEM and TEM pictures of as-etched GaAs/AlGaAs nanopillars by ultimate top-down process. (a) bird's eye view, (b) cross-section, and (c) TEM image after AlGaAs regrowth.

Figure 2(a) and 2(b) show SEM images of the as-etched pillars. The images in Fig. 2(b) clearly show that high-aspect-ratio nanopillars (diameter <20 nm and height = 100 nm) were obtained by Cl_2_–NBE using metal oxide core masks. After etching, clear lattice images were visible on the sidewalls, suggesting that no significant critical physical damage had occurred. The surface of the nanopillars was then passivated by hydrogen-radical treatment at room temperature to prevent surface oxidation. To confirm the ND crystal quality, we also inspected a sample using cross-sectional high-angle annular dark field scanning transmission electron microscope (HAADF-STEM). Figure 2(c) shows HAADF-STEM images for checking the stacked structure of the NDs. MQWs of 8-nm-thick GaAs well and 4-nm-thick Al_0.275_GaAs barrier was grown and shaped into nanopillars, and then an Al_0.15_GaAs/GaAs cap was regrown to clearly observe the GaAs NDs that was embedded in a low Al concentration in the AlGaAs layer. Because of lattice-matched systems, it was difficult to clearly observe the NDs structure, and therefore the Al concentration was controlled between 0.15 at MQWs barriers and 0.275 at regrown barriers.

The time-resolved PL spectra of the fabricated structures were obtained. The samples were excited using second-harmonic ultra-short pulses with a time width of 150 fs from a mode-locked Ti:sapphire laser operating at a wavelength of 400 nm ( = 3.10 eV). The excitation density was 0.084 μJ/cm^2^, and the typical spot diameter on the sample surface was approximately 100 μm. PL was dispersed spectrally using a monochromator and detected using a streak camera. The sample was mounted on the surface of a low-temperature stage controlled using a closed-cycle helium cryostat, which enabled measurement of the temperature dependence of PL at temperatures ranging from 7 K to 200 K.

**Figure 3:**
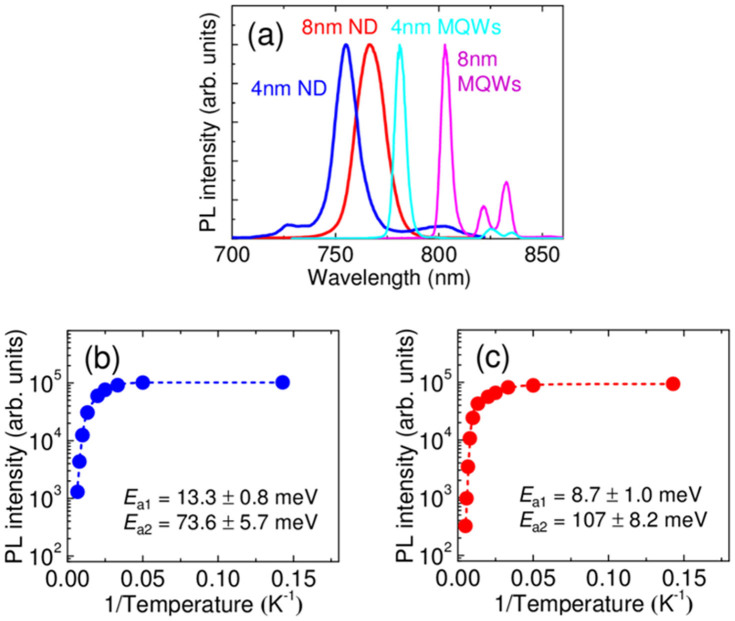
Temperature-dependent PL spectra of the (a) 4-nm-thick and 8-nm-thick GaAs quantum NDs and MQWs at 7 K. Integrated PL intensities of the (b) 4-nm-thick and (c) 8-nm-thick GaAs quantum NDs as a function of the inverse temperature.The dashed lines are fitting results obtained using Eq. (1).

Four different samples as respective reference materials were prepared and subjected to PL analysis: thin (4-nm-thick GaAs NDs) and thick ND (8-nm-thick GaAs NDs) samples and thin QW (4-nm-thick-QW) and thick QW (8-nm-thick-QW) samples. The time-integrated PL spectra of the thin and thick ND samples and the thin and thick QW reference samples at 7 K are measured in Fig. 3. The PL emissions from the thin and thick GaAs QW samples were observed at 1.62 eV ( = 764 nm) and 1.55 eV ( = 800 nm), respectively. In contrast, PL bands centered at 1.74 eV ( = 713 nm) and 1.66 eV ( = 747 nm) were observed for the thin and thick ND samples, respectively. The latter PL bands were clearly distinguished from those observed in the PL spectra of the GaAs QWs and were shifted to higher energies by 120 and 110 meV compared with those of the thin and thick GaAs QW samples, respectively. These significant shifts in the energies of the PL bands of the NDs were attributed to three-dimensional quantum confinement effects. A reduction in PL intensities was observed,because the volume of gain media shrank after realizing NDs. GaAs gain volume was reduced to 1/100 before MQWs after NBE, therefore, PL intensity degradation of NDs were as the same order of that of MQWs.

To gain insight into the optical properties of the new ND structures, the temperature dependence of the PL spectra of both the thin and the thick ND samples were measured over the range of 7 to 200 K, as shown in Figs. 3b and 3c, respectively. With increasing temperature, the PL intensities for both ND samples decreased monotonically, while the PL peak energies shifted to the lower energy region. The integrated spectral intensities of the thin and the thick ND samples were then semi-logarithmically plotted as a function of the inverse temperature [Figs. 3b and 3c, respectively], and similar behaviour was observed for both samples, with the PL intensity decreasing moderately down to 100 K and rapidly above 100 K. These temperature-dependent PL intensities suggest the presence of nonradiative recombination processes.

Next, the temperature dependence of the PL intensity was analysed using the following Arrhenius-type equation^19^,^20^,^21^.
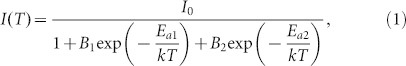
where *I*_0_ is the PL intensity at 0 K, *E*_a1_ and *E*_a2_ are the thermal activation energies for the PL-quenching processes, and *B*_1_ and *B*_2_ are coefficients relating to the number of nonradiative centers for the respective processes. The fitting results for the two ND samples with different thicknesses are shown in Figs. 3b and 3c. Here, the two exponential terms in eq. (1) are necessary to reproduce the experimental data, thereby indicating two different types of nonradiative recombination mechanisms.

**Figure 4:**
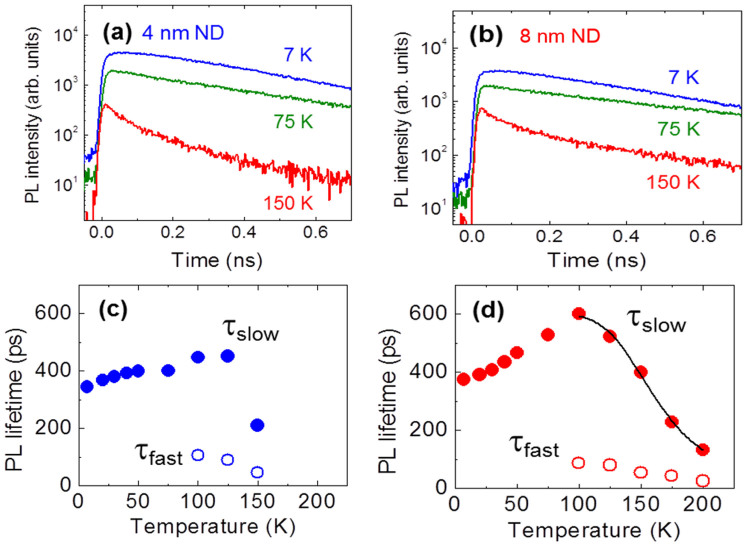
PL time profiles of the (a) 4-nm-thick and (b) 8-nm-thick quantum NDs as a function of temperature (7, 75, and 150 K), and (c) and (d) temperature dependence of the PL decay times for the respective samples in (a) and (b). The closed and open circles correspond to slower and faster PL decay times, respectively.

Time-resolved PL spectra of the samples at temperatures ranging from 7 K to 200 K were then observed in order to gain more insight into the carrier dynamics, including the nonradiative quenching processes. The time-resolved PL spectra were recorded using a synchroscan streak camera (Hamamatsu photonics, C4334) with a time resolution of approximately 5 ps after the deconvolution analysis. Typical PL time profiles of the thin and thick ND samples were measured at 7 K, as shown in Figs. 4(a) and 4(b). Below 100 K, the PL transients were well-fitted using a single exponential function, whereas the double exponential components were required for the fitting above 100 K. The decay times obtained are plotted as a function of temperature for the thin and thick ND samples in Figs. 4(c) and 4(d), respectively. The temperature dependence of the PL decay time for the two samples is similar, with a slower decay time that increases as the temperature increases up to 100 K and then decreases above 100 K.

To study the relationship between measured activation energies and ND structures, the ND energies were estimated using known parameters, such as the temperature dependence of semiconductor energy levels as determined using the Varshini empirical equation^22^, effective masses^23^, ND profiles, and measured optical transition energies. The ND confinement energies were calculated by solving Schrodinger's equation for the GaAs/AlGaAs finite potential well using the three-dimensional finite element method (FEM)^24^. The calculated band parameters are shown in Table 1.

## Discussion

As described in the Results section, the fitting coefficients for *B*_1_ determined to be 16 and 8.7 which is responsible for *E*_a1_, were found to be significantly lower than those for *B*_2_ determined to be 1.86 × 10^4^ and 9.69 × 10^4^ for the thin and thick ND samples, suggesting that the nonradiative process in the low-temperature regime plays a minor role in determining the overall PL behavior of the NDs. Therefore, the origin of activation energy *E*_a2_ was explored.

The larger activation energies *E*_a2_ were determined to be 73.6 and 107 meV for the thin and thick ND samples, respectively. These activation energies explain the rapid quenching of PL above 100 K and can be attributed to the effective barrier height for thermal leakage of excitons or carriers from the confined ND states. This conclusion is supported by the fact that *E*_1a_ for the thin ND sample was lower than that for the thick ND sample because of the stronger confinement and resulted in quantum states of higher energies.

The energy differences (187 meV for the thin ND sample and 248 meV for the thick ND sample) between the transition energies of the confined states (corresponding to the PL emission peaks) and the band gap energy of the AlGaAs barrier were much larger than the obtained activation energies. Therefore, thermal escape of excitons cannot account for the nonradiative process. In addition, for the thin ND sample, the calculated barrier height for an electron at the ground state was 99 meV and that for a heavy hole was 88 meV, while the experimentally obtained value of the activation energy *E*_a2_ was 73.6 ± 5.7 meV, which was close to the barrier height calculated for the valence band rather than that for the conduction band. For sample B (the thick ND), the calculated barrier height for an electron was 145 meV and that for a heavy hole was 103 meV. Therefore, the *E*_a2_ value of 107 ± 8.2 meV was again well in accordance with the barrier height for the valence band. Based on these results, we concluded that the nonradiative recombination process described by the activation energy *E*_a2_ can be attributed to the thermal escape of heavy holes from the confined ground states to the valence band of the AlGaAs barrier.

The results of the time-resolved PL measurements for the thick ND sample further confirmed this hypothesis for the thermal quenching of PL in the QDs. The PL decay time decreased at 100 K, which coincided with rapid decrease in the PL intensity beginning at 100 K. The observed temperature dependence of the PL decay time above 100 K for the thick ND sample was analysed using a similar Arrhenius-type equation, as follows^21^,^25^.
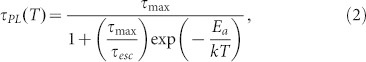
where τ_max_ is the maximum decay time at 100 K, *E*_a_ is the activation energy, and τ_esc_ is the escape time of the carriers from the ND states to the barrier. From the fitting calculations, the activation energy *E*_a_ was found to be 97 ± 5 meV, with an escape time τ_esc_ of approximately 0.6 ps. The value of *E*_a_ deduced from the PL decay time was close to that obtained from the temperature dependence of the PL intensity (approximately 107 meV). This agreement again suggests that the nonradiative recombination process, which causes rapid decreases in the PL intensity and decay time above 100 K, can be attributed to the thermal escape of heavy holes from the confined ground states of ND to the valence band of the barrier.

We note that a faster decay was also observed in the high temperature range above 100 K, as shown in Figs. 4c and 4d. The time constants (τ_fast_) were similar for both ND samples; therefore, this fast process can be attributed to the extrinsic, rather than intrinsic, nature of NDs. In addition, the presence of this fast decay indicates that, in some sample areas, nonradiative recombination centres may exist in the barrier at the GaAs–AlGaAs interface of the sidewalls of NDs. Thermally excited carriers may be rapidly trapped by nonradiative centres in the barrier with high impurity level of oxygen and carbon were derived from the etching process that were still existed between the interface of NDs and regrown barrier, which then exhibit fast decay.

The lower activation energies (*E*_a1_) were determined to be 13.3 and 11.1 meV for the thin and thick ND samples, respectively. These activation energies describe the moderate decrease in the PL intensity at temperature above 75 K. The values of *E*_1a_ agreed well with the energy difference between the ground and first excited states (eigenvalues) of the valence bands (*E*_hh_^2^ − *E*_hh_^1^), which were calculated to be 12 and 11 meV for the thin and thick ND samples, respectively. Therefore, the nonradiative process described by *E*_1a_ is attributed to the thermally excited excitons (*E*_e_^1^ − *E*_hh_^2^) as an optically inactive dark state with slightly higher energy^26^. The overlapping integral of the wave functions of the ground-state electron and the first excited heavy hole is nearly zero in the present calculation. Thereforethe oscillator strength of this electron–hole pair is also nearly zero, thereby indicating that the optically inactive exciton states are nonradiative. The low difference in the value of *E*_a1_ for the 2 samples may be explained by the difference in the strength of the quantum confinement; when the size of the ND decreases, the energy interval of the confinement states increases.

This assignment of *E*_a1_ is also consistent with the temperature dependence of the PL decay times. In general, the radiative recombination time is determined by the net and effective oscillator strengths of the ND, reflecting the density of states and thermal distribution of the excitons^27^,^28^,^29^. In a stronger quantum confinement regime, because the effect of the thermal excitation at higher energy-confined states is significantly reduced due to the discrete energy separation, the net oscillator strength nearly equals that of the ground state, and the radiative recombination time remains constant with increasing temperature. When the quantum confinement is not strong enough, the excitons can populate the higher energy states that have lower oscillator strengths as the temperature increases, resulting in a gradual increase in the radiative recombination time with increasing temperature. For the thin ND sample, the PL decay time moderately increased from 350 ps at 7 K to 440 ps at 100 K. On the other hand, in the thick ND sample, the PL decay time significantly increased from 380 ps at 7 K to 600 ps at 100 K. Therefore, the incremental increase in the PL decay time with increasing temperature in both samples can be attributed to thermal excitation of excitons into optically inactive exciton states with lower oscillator strengths. The significant increase in the PL decay time for the thick ND sample compared with the moderate increase for the thin ND sample can be explained by the difference in the strengths of the quantum confinements. In the thin ND sample, the density of states is more discrete, thus, the energy separation is greater, preventing thermal excitation to the higher optically inactive states. As a result, the PL decay time is insensitive to the temperature for the thinner NDs. This thermal excitation to the optically inactive states is also consistent with the moderate decrease in the PL intensity with a low activation energy (*E*_a1_).

**Figure 5:**
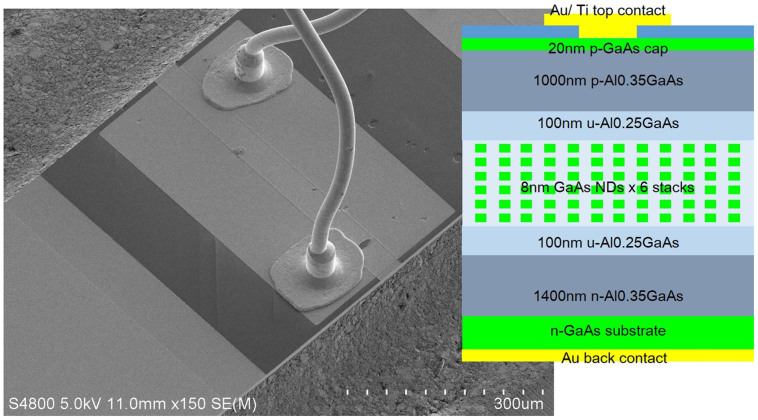
SEM image of top-down fabricated ND-LED.

After the regrowth process, we made an LED structure as shown in Fig. 5. First, we deposited a 300-nm-thick SiO_2_ layer and opened a window of 20 μm to 100 μm wide. Next, we deposited a 20-nm-thick Ti and a 300-nm-thick Au to make the top electrodes. Third, lapping of the GaAs substrate was performed to a thickness of 150 μm, and a 20-nm-thick AuGe and a 100-nm-thick Au was deposited on the rear side. For the LED emission experiments, we cleaved the sample into a bar piece without a subsequent coating process on the edge.

**Figure 6:**
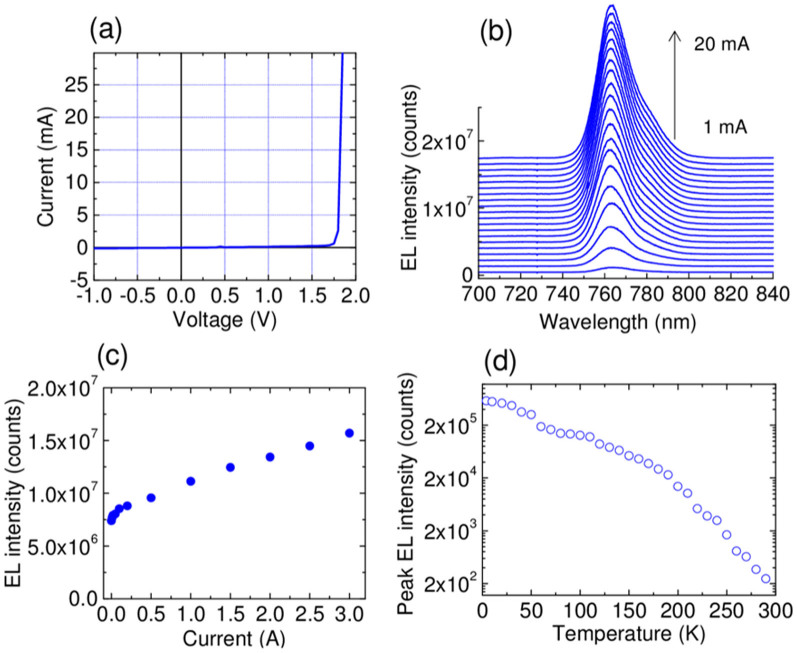
I-V and I-L characteristics of GaAs ND-LED. (a) I-V characteristic, (b) ND-LED emission spectrum, and (c) I-L characteristics of ND-LED. (d) Temperature dependence of ND-LED.

To induce device excitation, we used a continuous wave current source. The optical emission normal to the cleaved surface was detected by a spectrometer equipped with a charge-coupled device. Figure 6(a) shows the I-V curve of the ND-LED operated at 7 K. The width of the stripe electrode was 20 μm, and a threshold voltage of 1.8 V was observed. The ideality factors were approximately 2 between 1.75 V and 1.85 V. This indicates that the carrier recombination at the junction was dominant^30^. Figure 6(b) shows the emission spectrum taken from the cleaved edge of the LED structure for various current injection conditions at 7 K. The spectra show a smooth and narrow curve, corresponding to the emissions from the ND ensemble. The center energy of the line ensemble was 763 nm, which agreed well with that of the ND emissions. This observation suggests that the emission was originated from the ground state of the NDs, which was attributed to the formation of the high-density NDs at high uniformity. Figure 6(c) plots the emission intensity profile measured at various current values at a 10 kHz pulse current injection. An offset emission was observed at an offset direct current (DC) of 4 mA. As pulse current was increased, emission power increased linearly as usually seen in a typical LED behavior. The temperature dependence up to room temperature of the EL intensity of ND-LED with higher Al-content (30%) in regrown AlGaAs layer. In the original sample, the barrier material was Al_0.15_Ga_0.85_As, and thus the intensity of EL at room temperature was not enough for the detection. This was due to the relatively lower barrier height of Al_0.15_Ga_0.85_As, and that the majority of injected carriers were thermally flown out from the NDs; it was also predicted by the calculation. To solve this problem, we used a higher aluminum content in AlGaAs for regrowth, and prevented the thermal escape. According to figure 6(d), we observe that the room temperature operation of GaAs ND LED is possible.

In summary, we demonstrated the current-injection operation of a GaAs ND-LED developed by the ultimate top-down process and MOVPE regrowth. By using the damage-free NB etching, we achieved high-density GaAs/AlGaAs QDs at high uniformity and quality, thereby realizing an intense narrow PL emission from the ensemble of NDs. The ND-LED structure of these NDs exhibited a narrow emission with a clear threshold at 7 K. The strain-free GaAs/AlGaAs system has a definite advantage in enabling a large number of QD layers, while keeping a high crystallographic quality since there is no strain-induced dislocation. Therefore, this developed manufacturing process is thought to be a promising method to produce high-performance ND optical devices in the lattice-matched compound semiconductor systems for yet further optimization.

## Author Contributions

A.H. and T.K. performed experiments, simulations and prepared the manuscript text and all figures. C.T., Y.T., J.T., Y.W. and H.S. performed experiments. A.H., M.S. and Y.N. designed the experiment. I.Y., A.M. and S.S. edited the manuscript. All authors reviewed the manuscript.

## Figures and Tables

**Figure 1:**
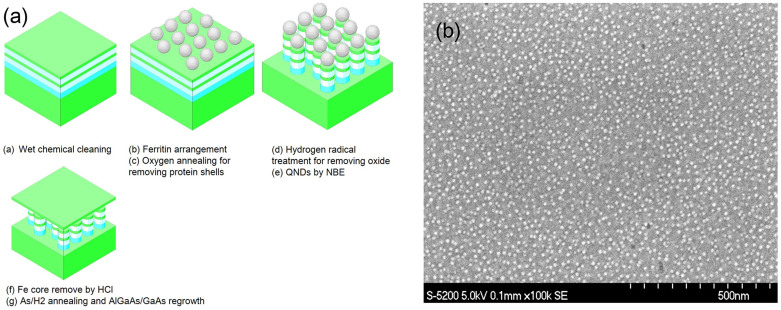
GaAs QNDs fabrication process and Ferritin arrangement after spincoat. (a) Process chart (b)SEM picture of after ferritin arrangement.

**Table 1:** Three-dimensional FEM simulated and measured activation energies of the unstrained GaAs/Al_0.275_GaAs quantum ND structure at 7 K

	Thin ND (4-nm-thick ND)	Thick ND (8-nm-thick ND)
GaAs bandgap level E_GaAs _[eV]	1.519	
Al_0.275_GaAs bandgap level E_AlGaAs _[eV]	1.865	
Ground state electron confinement energy E_e_^1^ [meV]	126	80
First excited state electron confinement energy E_e_^2^ [meV]	176	134
Ground state heavy hole confinement energy E_hh_^1^ [meV]	29	15
First excited state heavy hole confinement energy E_hh_^2^ [meV]	38	24
E_hh_^2^ − E_hh_^1^ [meV]	9	9
Transition energy	1.674	1.614
E_T_ = E_e_^1^+E_hh_^1^ + E_g _[eV]		
Activation energy for valence band [meV]	99	145
Activation energy for conduction band [meV]	92	106
Measured activation energy [meV]	73.6 ± 5.7	107 ± 8.2

Energy of conduction band offset: Energy of valence band offset = 0.65:0.35.
